# DNA methylation and demethylation in adipocyte biology: roles of DNMT and TET proteins in metabolic disorders

**DOI:** 10.3389/fendo.2025.1591152

**Published:** 2025-06-20

**Authors:** Lijiao Wu, Xiangjin Wang, Luling Wang, Shuyan Li, Qiu Chen

**Affiliations:** ^1^ Department of Endocrinology, Hospital of Chengdu University of Traditional Chinese Medicine, Chengdu, China; ^2^ School of Sports Medicine and Health, Chengdu Sports University, Chengdu, China; ^3^ Faculty of Chinese Medicine, Macau University of Science and Technology, Chengdu, China

**Keywords:** DNA methyltransferase family, ten-eleven translocation proteins, adipocyte biology, DNA demethylation, epigenetic mechanisms

## Abstract

Adipocytes play a crucial role in regulating energy metabolism throughout the body. Dysfunctional adipocyte biology is a primary factor in the development of metabolic disorders associated with obesity and type 2 diabetes. Over the past decades, the role of epigenetic mechanisms, particularly DNA methylation, in the development and regulation of adipocytes has been extensively elucidated. These mechanisms influence numerous biological processes in adipose tissue and adipocytes, including lipogenesis and lipid metabolism. With the discovery of the active DNA demethylation mechanism centered on ten-eleven translocation (TET) proteins, a growing body of evidence sug-gests that DNA demethylation mechanisms also profoundly influence various aspects of adipocyte biology and regulate cellular differentiation and function by altering the methylation status of genes. Following the discovery of active DNA demethylation mechanisms mediated by TET proteins, a growing body of evidence indicates that these mechanisms profoundly influence multiple aspects of adipocyte biology. Specifically, these mechanisms regulate cellular differentiation and function by altering the methylation status of key genes involved in adipogenesis and metabolism. A precise and detailed understanding of the mechanisms underlying DNA demethylation in adipocyte biology is imperative for the identification of novel interventional therapies targeting adipocyte gene methylation and demethylation. This review examines the specific molecular mechanisms and significance of passive and active DNA demethylation in adipocyte biology, focusing on the DNA methyltransferase family and TET proteins. It summarizes crosstalk mechanisms involving DNA methyltransferases, highlights the multiple action pathways of TET proteins, and reveals the potential of additional intervention pathways. This review aims to provide an updated theoretical basis for promising therapeutic targets.

## 1 Introduction

Since its initial identification in bacteria in 1925, DNA methylation has been the subject of extensive investigation across a diverse range of organisms and is currently the most intensively studied epigenetic mechanism (1). In mammals, DNA methylation typically occurs at cytosine-guanine dinucleotide (CpG) sites and mediates gene expression silencing in promoter regions. This process relies on a family of DNA methyltransferases (DNMTs) to catalyze the transfer of a methyl group from S-adenosylmethionine (SAM) to the fifth carbon of a cytosine residue, resulting in the formation of 5-methylcytosine (5mC). While this modification does not affect the base pairing of cytosine, it can alter the functional state of regulatory regions, thereby exhibiting the classic “epigenetic” marks. DNA methylation plays a crucial role in maintaining genomic stability, genomic imprinting, and chromatin structure (2–5). Consistent with these significant roles, a growing number of human diseases have been shown to be associated with DNA methylation including lipid metabolism disorders such as obesity, which was first linked to this epigenetic process over a decade ago (6). From the initial identification of genome-wide methylation loci associated with adipocyte differentiation in 2008 to recent comprehensive methylome analyses of adipocytes, a series of studies has demonstrated that DNA methylation is widely present in adipocytes and influences adipogenesis, adipocyte differentiation, and adipocyte function (7–9).

Acting as key endocrine and secretory cells, adipocytes are extensively involved in diverse physiological and metabolic processes through the synthesis and secretion of numerous protein signals and bioactive factors (10). Mammalian adipocytes comprise two major types: brown and white adipocytes. Brown adipocytes generate heat by oxidizing substrates such as glucose and fatty acids in response to diverse stimuli. White adipocytes store and release energy in the form of fatty acids in response to systemic energy demands (11). Another type of brown adipocyte that occurs in white fat depots was identified as a new type of adipocyte-beige adipocytes. Unlike classical brown adipocytes, beige adipocytes possess the ability to switch between energy storage and energy dissipation phenotypes (12). Research in physiology and pathology has revealed that adipocytes not only serve as regulators of systemic energy homeostasis but also that abnormal lipogenesis and lipid metabolism are key factors leading to dyslipidemia, obesity, fatty liver, and other diseases (13). The ongoing advancement of epigenetics has led to the growing recognition that a range of biological processes, including adipocyte function, lipid metabolism, and lipogenesis, are significantly influenced by DNA methylation. Neonatal mice show high levels of DNA hypermethylation in white adipose tissue (WAT) early in life and low levels in brown adipose tissue (BAT) (14). The combined impact of DNA hypermethylation and high-fat diet (HFD) feeding has been shown to profoundly disrupt abnormal adipocyte biology in mice and contribute to the development of diseases such as atherosclerosis in male mice (15–17). In light of these findings, de-(hypo)methylation therapies have been proposed as a potentially efficacious means of reversing the biological methylation process in aberrant adipocytes and of responding to changing metabolic environments, such as obesity, through altered metabolic gene methylation status. In fact, before the elucidation of the active DNA demethylation mechanism, DNA de-(hypo)methylation therapies had already been shown to significantly influence the pathogenesis of diseases such as cancer and atherosclerosis (18). A study conducted in 2009 demonstrated that DNA methylation inhibitors markedly diminish the capacity of 3T3-L1 cells to undergo adipogenesis during the contact inhibition phase (19). This suggests that targeted inhibition of DNA methylation at specific genomic segments plays a critical role in adipocyte biology. Following the elucidation of the mechanism of active DNA demethylation, this reversible methylation process and active DNA demethylation mechanism are regarded as potential avenues of intervention in a number of diseases.

**Figure 1:**
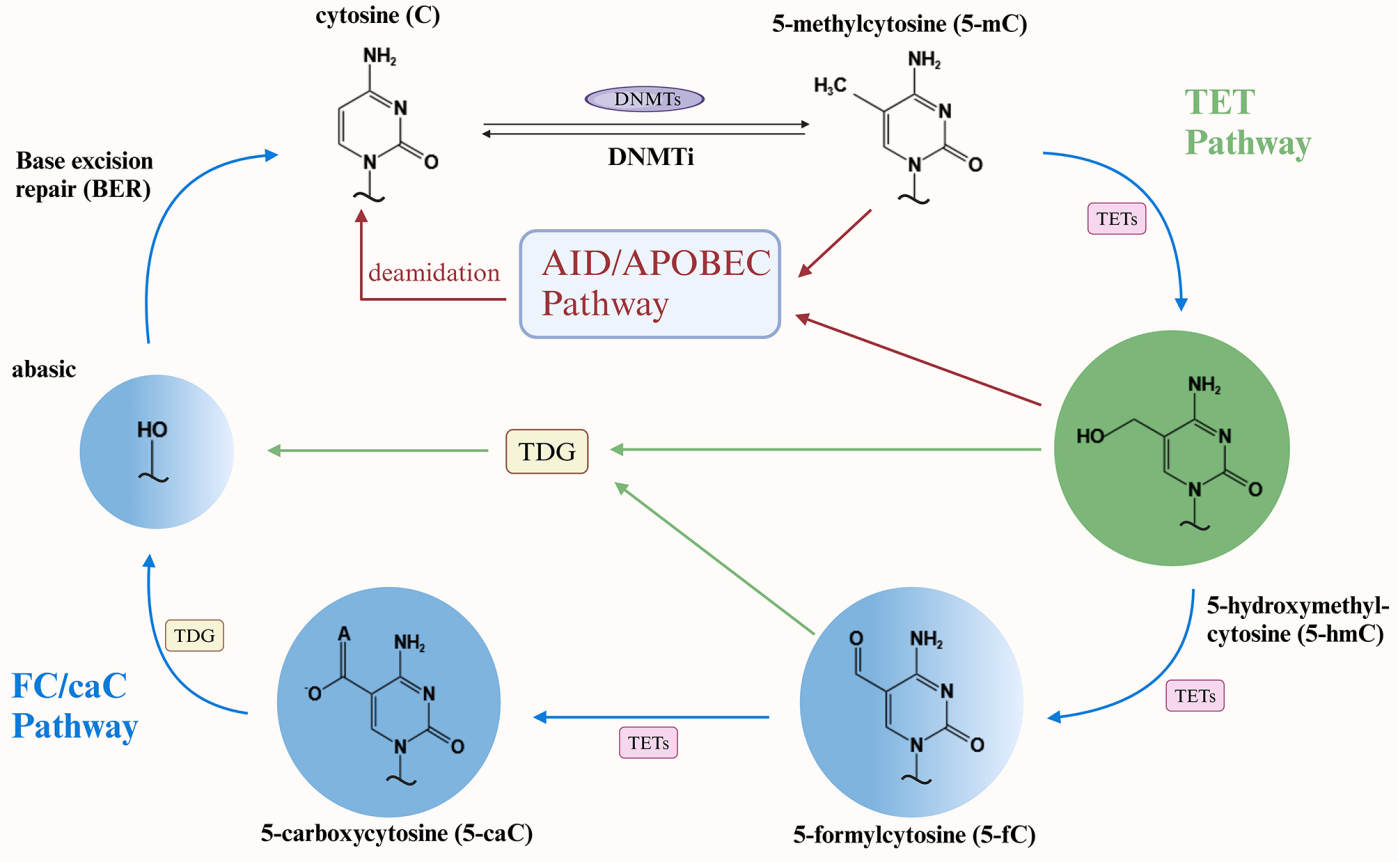
Diagram of DNA demethylation mechanisms. DNMTs, DNA methyltransferases; DNMTi, DNA methyltransferase inhibitors; TET, Ten-eleven translocation; TDG, Thymine DNA glycosylase.

The phenomenon of DNA demethylation was initially observed as a passive process. In an early study on embryonal carcinoma cells, it was demonstrated that a substantial reduction in DNA methylation (approximately 30%) upon retinoic acid induction facilitated cell differentiation (20). Subsequent studies have extensively elucidated the passive demethylation process, confirming that this link is closely associated with the failure to maintain DNA methylation and highlighting the dominant role of reduced or inhibited DNMT enzymes activity (21, 22). The introduction of three nuclear reprogramming methods in 2010 represented a significant shift in the decades-long understanding that DNA methylation is critical for maintaining stable cellular identity and advanced active DNA demethylation (23). Although the mechanism of active DNA demethylation has long been well characterized in plants, researchers initially failed to explore the mammalian counterparts to the plant demethylases, which placed mammalian active DNA demethylation in a state of uncertainty (24). It is notable that the initial identification of a mammalian DNA demethylase occurred as late as 1982. However, this discovery has following been overlooked or subjected to significant debate in the subsequent decades (25, 26). It is encouraging to note that a report on the conversion of 5mC from mammalian DNA to 5-hydroxymethylcytosine (5hmC) by human ten-eleven translocation (TET) one has provided a new perspective, suggesting the importance of 5hmC as an intermediate. In light of these findings, subsequent studies have corroborated that protein-mediated hydroxymethylation by TET, deamidation by the AID/APOBEC family, and base excision repair (BER) collectively represent the comprehensive mechanism underlying active DNA demethylation (27–30). Despite the recent identification of the active DNA demethylation mechanism, a series of comprehensive studies has provided a more detailed explanation of the underlying process. Overall, the active DNA demethylation process is achieved by the progressive oxidation or deamination of 5mC, which involves the following three pathways:(1) TET proteins successively oxidize 5mC to form 5hmC, 5-formylcytosine (5fC), and 5-carboxycytosine (5caC), which are then recognized and processed by the thymine DNA glycosylase (TDG) to initiate BER, constituting the canonical TET-TDG-BER pathway; (2) TDG-BER-independent, direct deformylation of 5fC and direct decarboxylation of 5caC; (3) AID/APOBEC family directly mediates 5mC or 5hmC deamidation (31–34) (**Figure 1**). Among these three pathways, given the recent discovery of direct deformylation of 5fC and decarboxylation of 5caC, as well as the re-evaluation of AID/APOBEC family-mediated deamidation, which evolved from early recognition, through scientific questioning, to re-establishment as a distinct mechanism in 2017 [detailed description in (32, 35)]. Consequently, the TET protein-mediated active DNA demethylation mechanism has been the most extensively studied among these three pathways. Previously published reports have highlighted the pivotal role of TET proteins in numerous biological processes and confirmed their importance as critical regulatory targets in diseases such as cancer (36). It is regrettable that, despite the publication of several significant studies in recent years that have elucidated the mechanisms and implications of TET proteins in adipocyte biology, a comprehensive summary of these findings has yet to emerge. The few existing reports on the relationship between DNA demethylation and lipid biology are limited in scope and depth, with a paucity of detailed mechanisms and a tendency to become obsolete. Accordingly, by concentrating on DNMT enzymes and TET proteins as the central focus, this review provides a detailed and in-depth summary of the mechanisms of passive and active DNA demethylation in adipocyte biology for the first time. It aims to further illustrate the significant role of epigenetics in adipocyte biology and hopes to offer deeper research targets for lipid regulation, adipogenesis, and lipid metabolism-related diseases, as well as promising clinical prevention and treatment pathways for the future.

## 2 DNMT family: taking control of passive DNA demethylation

The human genome encodes five DNMT: DNMT1, DNMT2, DNMT3A, DNMT3B, and DNMT3L. Among them, DNMT1, DNMT3A and DNMT3B are classical cytosine-5 DNMTs. They comprise an N-terminal regulatory domain and a C-terminal catalytic domain, utilizing SAM as a methyl donor and a base-flipping mechanism to translocate the target base into the catalytic pocket. This process ultimately results in the formation of 5mC via a methylation reaction (37). DNMT1 preferentially targets hemimethylated DNA within the methylation pathway to maintain the methylation pattern during DNA replication, while DNMT3s primarily act on unmethylated DNA substrates. This functional dichotomy has long supported the hypothesis that DNMT1 serves as the primary methylome maintainer, whereas DNMT3 enzymes facilitate *de novo* methylation (38, 39). Subsequent studies have further complemented the mechanism of maintenance of DNA methylation by demonstrating that recognition of hemimethylated DNA by DNMT1 is involved in the maintenance process in conjunction with localization of specific chromatin regions containing methylated DNA by the DNMT3s enzyme (40). Thus, DNMT1, DNMT3s dominate the passive demethylation process associated with failure to maintain DNA methylation. In lean murine adipocyte biology, silencing of DNMT1 accelerates adipogenic differentiation, while the expression of DNMT3A is significantly upregulated in the adipose tissue of obese mice. These findings suggest that DNMTs are intimately linked to adipocyte and adipose tissue (41, 42).

### 2.1 Overexpression of DNMT: mediating a bidirectional crosstalk mechanism

The degree of methylation of specific genes, which depends on the enzymatic activity of DNMT, affects adipocytes, and overexpression of DNMT a critical contributor to this regulatory process. Animal experiment data indicate that DNMT3A overexpression stimulates the proliferation and inhibits the adipogenic differentiation of porcine intramuscular preadipocytes. Studies have shown that the overexpression of DNMT1 and DNMT3A has the opposite effect on lipogenesis in 3T3-L1 cells, promoting and inhibiting the process, respectively, during the pre- and late-stage differentiation phases (8, 43). Furthermore, it was demonstrated that mice fed a HFD exhibited an increased number of hypermethylated regions and significant upregulation of DNMT3A expression; a significant increase in the methylation level of the leptin CpG promoter was accompanied by an associated increase in DNMT3A (44, 45). HFD significantly altered the enzymatic activity and global DNA methylation status of DNMTs in the gonads of mice and increased the levels of DNMT1 and DNMT3A proteins in the ovaries and testes (46). These studies highlight that excessive fat intake and the lipid metabolic microenvironment significantly affect DNMT enzymatic activity. Data from obese patients indicate that DNMT1 is highly expressed in both visceral and subcutaneous adipose tissue and exhibits a positive correlation with body mass index (BMI) (47). Notably, DNMT1 and DNMT3A, highly expressed in individuals with obesity, contribute to the induction of obesity-associated inflammatory responses. Conversely, inflammatory factors promote DNMT1 activity in adults with obesity, thus suggesting a bidirectional interaction between DNMT enzymatic activity and the inflammatory response in the obese microenvironment (48, 49). In addition, adipose tissue and cell type-specific gene expression profiles of subcutaneous fat from six female subjects were retrieved from adiposetissue.org (50). The profiles revealed that DNMT1 and DNMT3A were highly expressed in CD8+ T cells and M2 macrophages, respectively. This finding supports the involvement of DNMTs in the adipose tissue inflammatory response. Similarly, DNMT enzymatic activity exhibits bidirectional crosstalk with adipocyte biology, and hypermethylation of specific genes, mediated by DNMT overexpression, significantly influences lipid metabolism and regulation. DNMT1 is hyperactivated in adipocytes of obese subjects. Activated DNMT1 selectively methylates the promoter of adiponectin genes involved in lipid metabolism regulation, thereby inhibiting adiponectin expression (49). *In vitro* experiments demonstrated that high glucose-induced lipid accumulation occurs via inducing DNMT1-mediated DNA hypermethylation of specific genes. A key pathway involves certain oxysterols, which not only regulate lipid metabolism and inflammatory responses but also serve as agonists to promote DNMT1 expression (51).

### 2.2 DNMT inhibition: passive demethylation and adipocyte biology

Although subsequent studies have elucidated DNA methylation maintenance mechanisms, research on DNMT1’s role in this process has been extensively studied, especially in the context of passive demethylation mechanisms in adipocyte biology. Previous *in vitro* experiments demonstrated that DNMT1 expression regulates the timing of adipocyte differentiation and confirmed that DNMT1 silencing accelerates this process (42). A study investigating the epigenetic regulator tonicity-responsive enhancer-binding protein (TonEBP) in thermogenesis and obesity found that treatment with the DNMT inhibitor RG108 (12 mg/kg, intraperitoneal injection every two days) conferred resistance to HFD-induced body weight gain and adiposity in mice, confirming that DNMT1 primarily mediates this protective effect (52). The paper by Park and colleagues presents a compelling argument that DNMT1 is the most abundant DNA methylation modifier in adipose tissue, and that adipocyte DNMT1 is required for the maintenance of the obese phenotype and systemic energy homeostasis. Using adipose-specific DNMT1 knockout mice, the authors demonstrated that adipocyte DNMT1 deficiency promotes lipid accumulation via promoter hypomethylation, exacerbates obesity-induced impairments in adipose tissue remodeling and energy metabolism, and induces hypertrophic expansion of adipose tissue (53). In addition to this, recent studies have supported the finding that DNMT3A and DNMT3B deletion plays a pivotal role in adipose biology. A study on heterozygous DNMT3A deletion in mice demonstrated that DNMT3A-deficient mice exhibited a hypomethylated landscape, characterized by an inflammatory phenotype in adipocytes, an overall metabolic manifestation of obesity and age-related insulin resistance, and a broader spectrum of aberrantly differentiated adipocyte progenitors (54). Paradoxically, Dnmt3B knockout mice exhibited reduced energy expenditure, susceptibility to diet-induced obesity and insulin resistance (55). A further intriguing study revealed that mice with hematopoietic DNMT3A deficiency exhibited more pronounced weight gain, a heightened inflammatory response, and glucose intolerance when subjected to the same HFD (56). These experiments not only confirmed that DNMT3A inhibition disrupts lipid metabolism regulation by mediating DNA demethylation, but also showed that DNMT3A plays an important role in inducing lipid metabolism-related inflammatory responses.

### 2.3 DNMT inhibitors: promising clinical treatments

More targeted research has emerged in the field of DNA methyltransferase inhibitors (DNMTi). Although the majority of DNMTi therapeutic effects are focused on oncology, numerous studies now explore their role in adipocyte biology. 5-Azacytidine (5AC) and decitabine (5-azido-2’-deoxycytidine, DAC) have become widely utilized in oncology research as common DNMTi since they were identified in the early 1980s for their ability to reverse DNA methylation (57–59). In adipocyte biology, the effect of 5AC on the differentiation of adipose-derived stem cells is well documented. Its inhibitory effect on methylation has also been demonstrated to disrupt cholesterol and lipid homeostasis. Treatment with DAC induces significant hepatic DNA hypomethylation in mice fed with HFD, thereby leading to a marked reduction in hepatic lipid accumulation (60–62). Moreover, both have been demonstrated to positively regulate leptin gene expression, which in turn regulates fat mass. In 3T3-L1 cells, 5AC-mediated DNMT1 inhibition promotes leptin expression via DNA hypomethylation; similar marked increases in leptin expression have been observed in decitabine (DAC)-treated fibroblasts and HeLa cells (63, 64).

**Figure 2:**
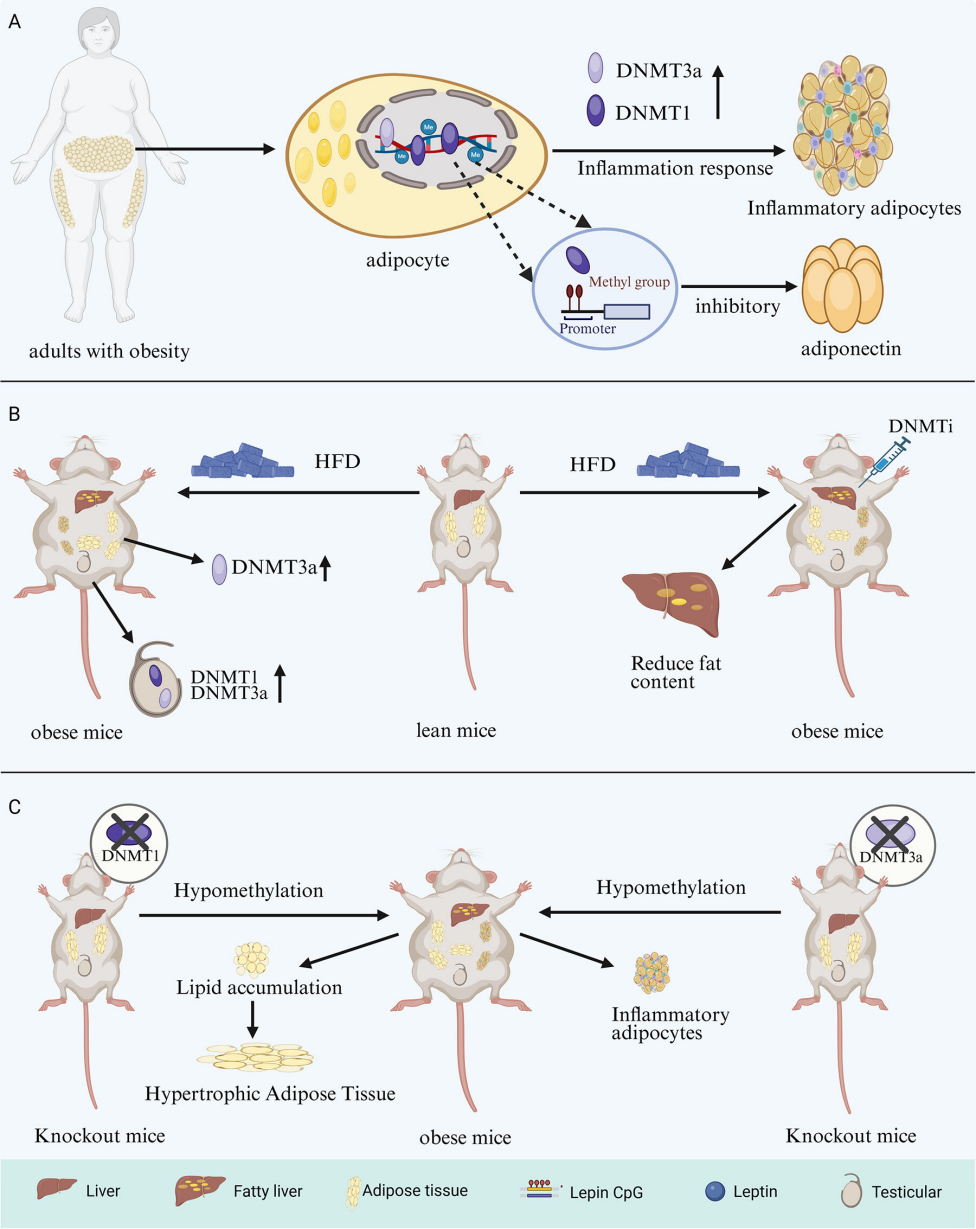
DNMTs and adipose biology-related mechanism diagram. **(A)** Overactivation of DNMT1/3a by adipocytes in adults with obesity elicits inflammatory adipose tissue accumulation via inflammatory response and inhibiting adiponectin expression by selective methylation of DNMT1. **(B)** In HFD-fed mice, adipose tissue DNMT3a and testicular DNMT1/3a levels increased significantly, while DNMTi reduced hepatic fat content. **(C)** DNMT1 and DNMT3a knockout mice exhibit lipid accumulation, hypertrophic adipose tissue, and inflammatory adipocyte production through hypomethylation, respectively. DNMTs, DNA methyltransferases; HFD, High-fat diet; DNMTi, DNA methyltransferase inhibitors.

Notably, inhibition-mediated DNA demethylation mechanisms, which target DNMT, have been associated with dysregulated adipocyte-related disorders. These findings provide potential targets for the treatment and prevention of related disorders. In an ovarian cancer cell migration study, the effect of DNMTi on fully differentiated adipocytes demonstrated that hypomethylating drugs may influence the tumor microenvironment and, consequently, reduce cancer cell metastasis. This provides a potential mechanism for how epigenetic modulation of adipocytes may reduce metastasis (65). Combined low-dose DAC treatment and pharmacological DNMT1 inhibition has been demonstrated to ameliorate type 2 diabetes mellitus and halt the onset and progression of non-alcoholic fatty liver disease (NAFLD) by targeting and modulating macrophage polarization in adipose tissue (66, 67). DNMT inhibition-mediated hypomethylation of lipogenesis-associated genes represents a potential mechanism by which bisphenol A exposure may induce hepatic lipid accumulation. This suggests that targeted epigenetic therapy for lipogenesis may represent a promising strategy for ameliorating hepatic lipid accumulation (68) (**Figure 2**).

## 3 TET proteins: active regulators of DNA demethylation

**Figure 3:**
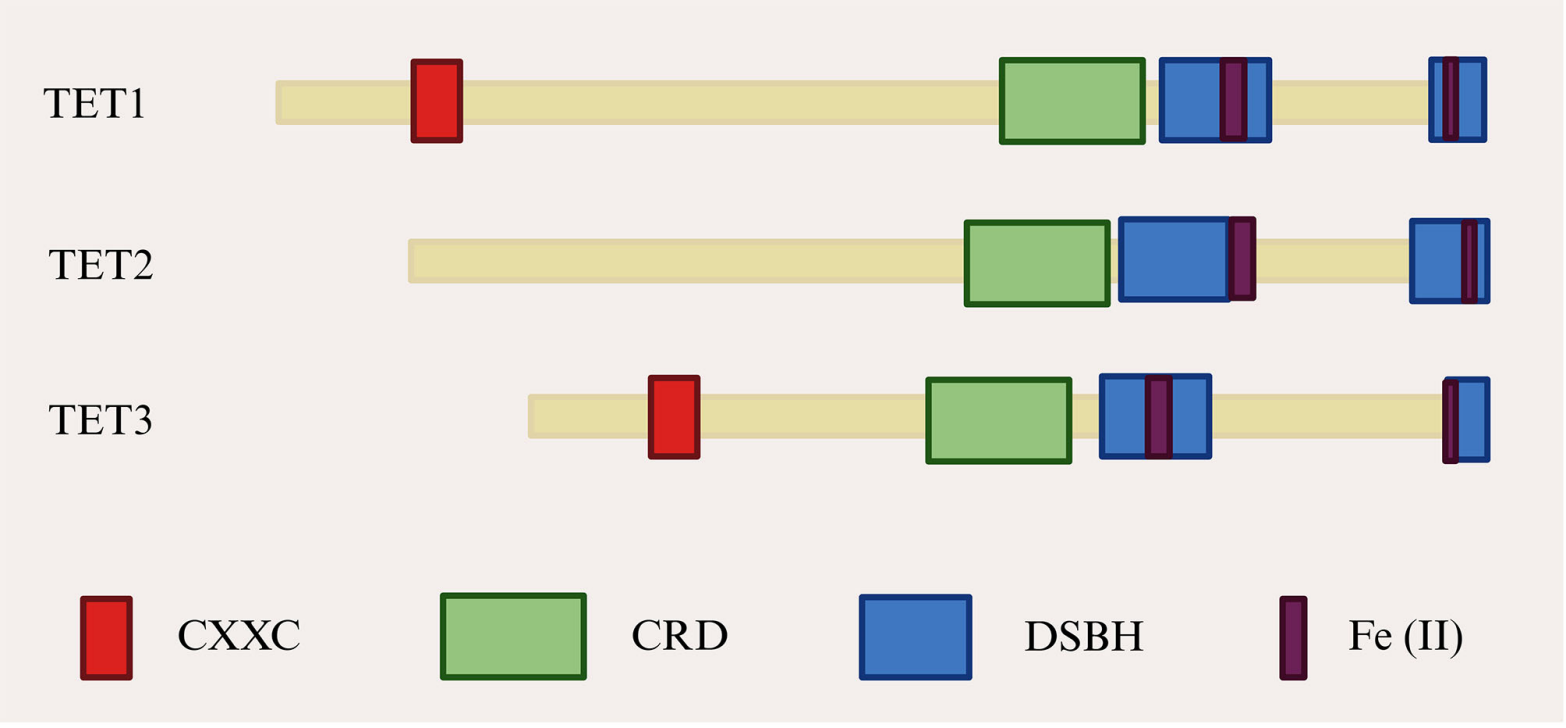
TET proteins structural domains. CRD, Cysteine-rich structural domain; DSBH, Double-stranded β-helical; TET, Ten-eleven translocation.

The first member of the TET protein family, TET1, was identified in patients with acute myeloid leukemia carrying the t (10;11) (q22; q23) translocation. Subsequently, TET2 and TET3 were identified and found to exhibit high sequence homology with TET1. (69, 70). TET proteins function as iron (II)/α-ketoglutarate (Fe (II)/α-KG)-dependent dioxygenases. Their primary structure includes a carboxyl-terminal catalytic domain, which contains a cysteine-rich domain (CRD) and two double-stranded β-helical (DSBH) regions (71). The amino-terminal regions of TET1 and TET3 contain a CXXC domain involved in CpG dinucleotide binding. In contrast, during evolution, the putative CXXC domain of TET2 was lost from the protein as a result of genomic inversion and was subsequently replaced by CXXC4 (36) (**Figure 3**). Owing to this structural variability, TET2 protein typically binds gene promoters indirectly, through interactions with transcription factors that modulate gene expression (72). Interest in TET proteins has centered on the observation that oxidized methylcytosine serves as an intermediate in DNA demethylation. TET-mediated oxidation of 5mC to 5hmC, 5fC, or 5caC represents a central pathway of active DNA demethylation. In-depth studies have demonstrated that active DNA demethylation, which is mediated by TET proteins, involves at least four processes: promotion of passive DNA demethylation, active DNA demethylation through DNA repair, enzymatic decarboxylation of 5caC, and dehydroxymethylation by DNMT enzymes [summarized in (73)]. This active DNA demethylation process occurs in diverse biological contexts and plays a significant role in the pathogenesis of neurological disorders and in oncology (74, 75). Consistent with these roles, the active DNA demethylation process, which is primarily mediated by the TET proteins, is also critical for the biology of adipocytes.

### 3.1 TET and adipocytes: in-depth epigenetic regulatory mechanisms

The TET protein family is critical for adipose tissue biology. TET1 has been identified as an epigenetic repressor of the beige adipocyte-selective thermogenesis gene program. It inhibits adipocyte thermogenesis in a cell-autonomous manner and exerts its effects in mice with diet-induced obesity (76). TET3, which shares high structural similarity with TET1, has been demonstrated to serve as a pivotal epigenetic regulator of homeostatic control of white adipose tissue deposition and diet-induced adipose expansion. It autonomously regulates adipogenic cells and is an indispensable protein for adipogenesis both *in vitro* and *in vivo* (77). TET2 with structural specificity has been identified as an anti-lipogenic demethylase in the differentiation of T3-L1 cells, and knockdown of TET2 profoundly impairs adipocyte differentiation, leading to a significant increase in lipogenesis (78). Notably, although the aforementioned studies demonstrated that TET1/2 can influence adipocyte biology independently of the DNA demethylation pathway, subsequent studies have corroborated the crucial role of TET proteins as DNA demethylation enzymes (76, 77). First, fundamental *in vitro* studies have systematically validated these findings. A study on the regulation of mesenchymal cell profiles by TET1/2 indicates that TET1 and TET2 serve as inhibitors and promoters of osteogenesis and adipogenesis, respectively, by mediating alterations in DNA demethylation status (79). TET3 functions as a DNA demethylating enzyme in conjunction with the pivotal transcription factor CCAAT/Enhancer Binding Protein δ (C/EBPδ) to promote adipocyte differentiation by catalyzing the DNA demethylation of C/EBP binding motifs and stimulating the expression of essential adipogenic genes (80). Findings from animal studies show that TET1 knockout is linked to downregulation of genes involved in lipid metabolism and adipocyte differentiation, and TET1 deficiency in mice fed with HFD has been observed to induce the upregulation of genes associated with lipogenesis and fatty acid uptake (81, 82). Decreased β3-adrenergic receptor (β3-AR) expression and blunted β-adrenergic signaling are strongly linked to obesity. Adipocyte-specific deletion of all three TET genes increases β3-AR expression, thereby enhancing lipolysis, thermogenesis, oxidative metabolism, and fat browning in transgenic mice. This effectively prevents obesity (83). Recent studies have demonstrated that TET2 acts as a DNA demethylase to regulate gene expression in human and mouse endothelial cells, and these studies have indicated that endothelial TET2 deficiency exacerbates HFD-induced obesity (84). These studies strongly indicate that the TET proteins play a pivotal role in regulating adipocyte biology. However, the interaction between these two factors is not a unidirectional process. More interestingly, HFD-induced obesity models have been demonstrated to diminish TET2 expression in adipose endothelial cells. Additionally, a recent study by Liu and colleagues revealed elevated muscle TET3 expression in humans and mice with obesity/diabetes, substantiating a robust positive correlation between obesity/diabetes and elevated muscle TET3 expression (84, 85). These findings suggest that the dysregulated lipid metabolic environment also influences TET protein expression. Consequently, selectively modulating TET proteins to promote or inhibit demethylation or methylation of specific genes may offer a promising strategy for correcting aberrant lipid metabolism.

### 3.2 PPAR: important mediation sites for TET proteins

Peroxisome proliferator-activated receptors (PPAR) are recognized as lipid sensors due to their capacity to regulate whole-body energy metabolism. The family consists of PPARα, PPARγ, and PPARδ, with PPARγ playing an important role in the regulation of adipogenesis and glucose metabolism, as well as promoting lipid storage and insulin sensitivity (86, 87). A genome-wide 5hmC analysis of differentiated adipocytes revealed elevated levels of 5hmC at the PPARγ binding site, suggesting that TET enzymes regulate adipogenesis by targeting the PPAR (88). Functional analysis of TET1/2 in adipogenesis showed that TET1/2 upregulation in mouse 3T3-L1 preadipocytes was accompanied by an increased expression of PPARγ. They observed that the knockdown of TET1/2 impairs adipogenesis by inhibiting PPARγ expression, thereby highlighting a more prominent role for TET2 in this process (89). The findings of Bian and Liu et al. further substantiate the dominant role of TET2. Their studies revealed, respectively, that TET2 promotes the mouse transcriptional activity of PPARγ in a catalytically dependent or independent manner and that TET2 deficiency blocks adipogenesis by repressing the expression of C/EBPβ, C/EBPα and PPARγ. Taken together, these findings suggest that TET2 deficiency is closely associated with adipogenesis (90, 91). Although the majority of studies have demonstrated an association between TET2 and PPARγ, it has been shown that the TET1 protein is also regulates adipocyte biology by targeting PPARα, which plays an important role in this process by increasing cellular fatty acid uptake, esterification, and transport, as well as regulating genes involved in lipoprotein metabolism (86). A study on NAFLD demonstrated significant reductions in PPARα and its downstream critical enzymes, including acyl-CoA oxidase 1 (ACOX1) and carnitine palmitoyltransferase 1A (CPT1A), as well as the fatty acid oxidation product β-hydroxybutyrate (β-HB), in TET1 knockout mice. This evidence supports the hypothesis that TET1 activates PPARα through hydroxymethylation. This activation promotes fatty acid oxidation and inhibits the progression of NAFLD (92). Intriguingly, studies focusing on NAFLD suggest that TET1 deletion may achieve a therapeutic effect in NAFLD mouse models through substantial downregulation of PPARγ expression. Additionally, another animal study revealed that TET2 plays a pivotal role in regulating the progression of NAFLD by mediating alterations in the methylation of C-Maf-induced proteins, which modulate the Gbp2-PPARγ-CD36 axis (93, 94). These studies demonstrate that TET proteins target PPAR nuclear receptors to regulate NAFLD progression by modulating adipose tissue biology. This suggests that DNA demethylation interventions targeting the TET protein pathway may be an effective method for regulating lipid metabolism-related diseases.

### 3.3 Targeting leptin: another important pathway of action for TET proteins

Leptin, the first identified adipokine to be identified, is a pivotal adipokine that facilitates adipose tissue-brain communication to sustain energy homeostasis and normal body weight (95, 96). It was initially hypothesized that leptin could act as a powerful hormone to promote weight loss in adults with obesity. However, elevated plasma leptin levels in individuals with overweight or obesity, together with leptin resistance as a major contributor to obesity pathogenesis, indicate altered leptin expression in obesity. These alterations have been potentially associated with methylation of its promoter region (97, 98). Prior research has demonstrated that the leptin promoter in the epididymal fat of diet-induced obese mice exhibits a markedly reduced methylation at week 8 compared with the low-fat group. This suggests that a high-fat diet may induce early DNA demethylation of the leptin promoter, leading to increased leptin expression (99). It is surprising that the study did not identify a significant inverse correlation between leptin promoter methylation and leptin transcription. Nevertheless, subsequent studies focusing on the relationship between TET proteins and leptin have provided further evidence of their association. Hypothalamic agouti-related peptide (AGRP)-expressing neurons are integral to the regulation of feeding behavior, and their activity is inhibited by leptin (100, 101). Xie et al. demonstrated that TET3 negatively regulates AGRP expression and influences leptin signaling in AGRP-expressing neurons (102). In their experiments, leptin was failed inhibit fasting-induced binge eating in TET3 knockout mice. These results suggest TET3 is required for leptin-induced suppression of AGRP expression in the cell lines. This highlights the critical role of TET3 in the central control of obesity. The latest research findings offer new insights and compelling evidence for the association between adipocyte TET2 and leptin (103). In this study, the authors treated primary differentiated adipocytes with various factors known to increase white adipose tissue concomitant with obesity and demonstrated that only leptin suppressed TET2 expression. They confirmed that TET2 deficiency ameliorates HFD-induced obesity and insulin resistance by partially decreasing leptin levels as well as that the expression of the leptin gene in adipocytes is regulated by TET2. Furthermore, analysis of human data provided evidence for a negative feedback loop between TET2 and leptin in the context of obesity.

### 3.4 Cofactors: important regulators for targeting TET in adipocytes

**Figure 4:**
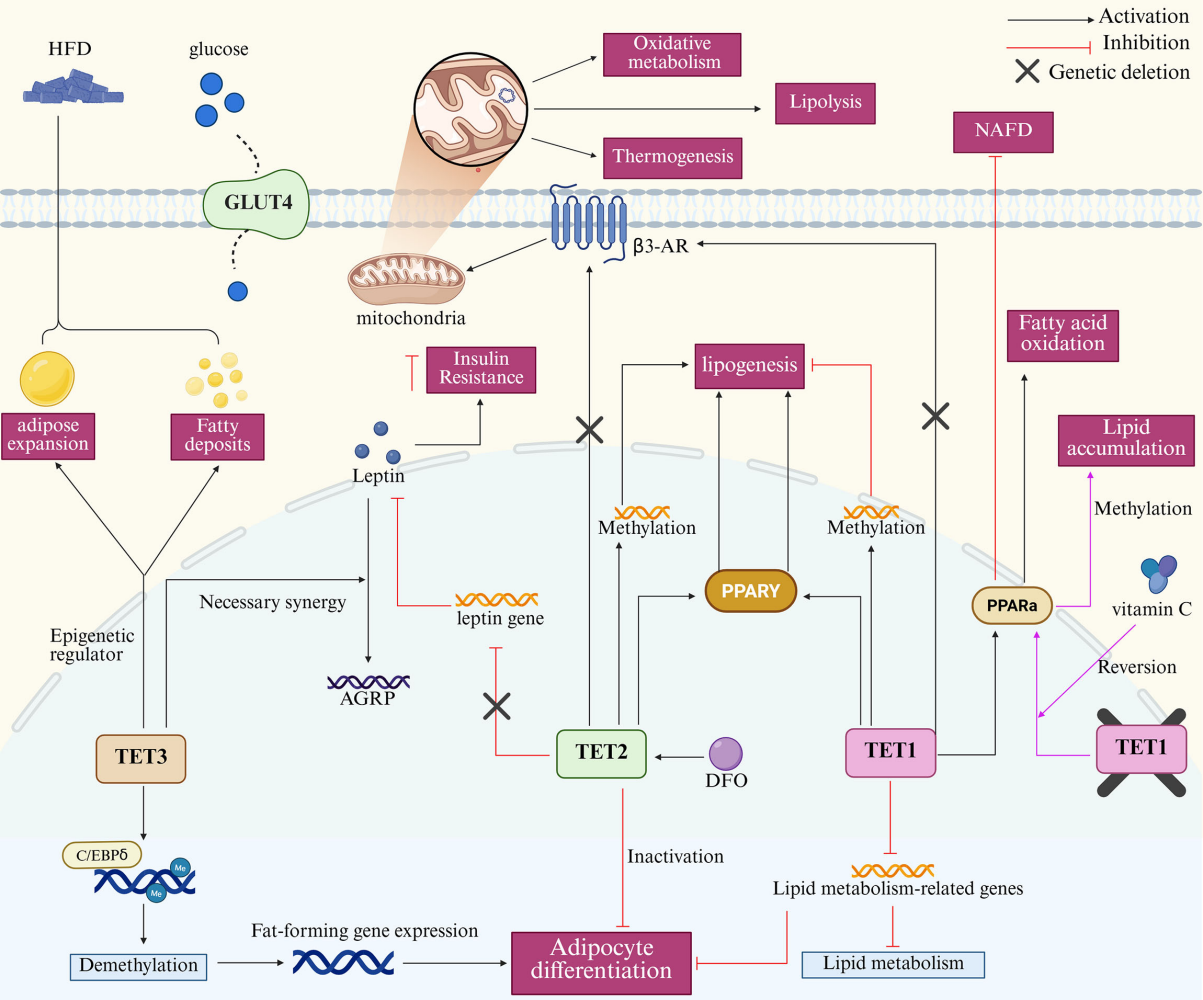
Mechanism of action of TET proteins in adipocyte biology. The core mechanisms in the map include TET3-mediated demethylation promoting adipocyte differentiation, adipose tissue expansion, and fat deposition. TET2 drives adipogenesis via methylation of PPARγ or related genes; TET2 deficiency lowers leptin levels. TET1 activates PPARγ/α to promote adipogenesis and fatty acid oxidation, respectively, and inhibit NAFLD progression. Deletion of all three TET genes upregulates β3-AR expression, enhancing mitochondrial lipolysis, thermogenesis, and oxidative metabolism. In adipose biology, vitamin C and DFO respectively promote and inhibit TET protein activity. TET, Ten-eleven translocation; HFD, High-fat diet; NAFLD, Non-alcoholic fatty liver disease; AGRP, Agouti-related peptide; C/EBPδ, CCAAT/Enhancer Binding Protein δ; DFO, Deferoxamine.

The structural properties of TET proteins and the requirement for key residues within the DSBH structural domain to bind cofactors—a necessary condition for optimizing catalytic function—collectively determine that cofactors are essential for the functional integrity of TET proteins (71). Iron is a canonical cofactor for Fe (II)/alpha-ketoglutarate-dependent dioxygenases (104). Studies of the iron-dependent effects of the TET proteins showed that TET2 in 3T3-L1 cells under deferoxamine (DFO) conditions had lower levels of 5mC, in marked contrast to untreated cells, confirming that TET2 mediates iron-dependent DNA demethylation during adipose differentiation (105). In addition, investigations into TET1-mediated active DNA demethylation in adipogenesis demonstrated that 0.1 mM DFO significantly suppressed lipid accumulation, indicating that iron-related TET inhibitors intervene in the adipogenic process (82). As a cofactor, vitamin C affects the function of TET proteins by promoting the folding of catalytic structural domains or iron recycling pathways (106). A critical role in determining the biological outcome of TET1 function has been reported for vitamin C (107). Mechanistic studies of TET1 in obesity revealed that vitamin C intervention improved the lipid metabolic status of TET1-deficient mice fed an HFD, reversed adipocyte hypertrophy in TET1 haploinsufficient mice, and effectively prevented Tet1-deficiency-induced lipid accumulation associated with methylation of the PPARα gene promoter (81). This study shows that TET1 plays a key role in lipid metabolism and suggests that vitamin C, acting as a cofactor, alleviates lipid metabolic dysfunction by enhancing TET1 activity. Therefore, targeting cofactors may also be an important way to regulate the methylation processes in the biology of adipose tissue. Unfortunately, the field currently lacks more in-depth research, and some important exploratory studies remain largely uncharted. For instance, whether cofactors influence TET protein activity in a dose-dependent manner and whether cofactors other than vitamin C exert beneficial effects on gene demethylation in adipocyte biology remain unresolved questions (**Figure 4**).

## 4 Other potential impact mechanisms

In addition to the extremely important role of TET proteins in the active DNA demethylation pathway of adipocyte biology, BER functions as a key component of the canonical TET-TDG-BER DNA demethylation pathway. The BER process is initiated by the DNA glycosylase, and TDG is one of the most representative DNA glycosylases in this process (108). Numerous studies have shown that TDG catalyzes the excision of 5mC, 5fC and 5caC produced by oxidation of TET proteins, and biochemical reconstitution assays of purified recombinant proteins have even demonstrated the existence of a direct interaction between TET1 and TDG. These findings highlight TDG’s essential role in the active DNA demethylation mechanism (109–113). TDG-mediated mechanisms in adipocytes involve PPARγ and fatty acid binding protein 4 (FABP4). In porcine preadipocytes, TDG depletion downregulated PPARγ and FABP4 mRNA expression, leading to a significant reduction in lipid droplet formation after induction of differentiation. The results indicate that inhibition of porcine preadipocyte differentiation by TDG knockdown is achieved by altering the methylation levels of some genes involved in adipocyte differentiation (114). Methyl-CpG-binding domain protein 4 (MBD4), a mammalian DNA glycosylase, suppresses mutations induced by 5mC deamination and excises guanine-mismatched uracil and modified uracil, and is a key player in ensuring the integrity of the active DNA demethylation process (115, 116). The discovery of the role of MBD4 in adipocyte biology preceded TDG. Studies on the differentiation process of porcine adipocytes confirmed the inhibitory effect of MBD4 on cellular differentiation, also by downregulating the mRNA expression levels of the cellular promoter regions C/EBPα, PPARγ, and adipocyte protein 2 (aP2) (117). Unfortunately, most current research on DNA glycosylases focuses on their BER function in the DNA damage response. In adipocyte biology, the specific roles and molecular mechanisms of DNA glycosylases in DNA demethylation pathways remain poorly understood.

It is also worth noting that the epigenetic regulation is a process that is susceptible to environmental and genetic factors, and the DNA demethylation process of adipocyte biology is no exception. This principle is illustrated by differences in DNA demethylation pathways in the adult male rat germline between HFD-induced and genetic obesity models (118). According to the Developmental Origins of Health and Disease (DOHaD) concept, maternal obesity and accelerated neonatal growth predispose offspring to WAT accumulation (119). Adult rat offspring of dams fed a HFD (termed HF) show adipocyte hypertrophy, hyperleptinemia and increased leptin mRNA levels in a depot-specific manner. These phenotypic changes correlate strongly with early-life modifications of epigenetic markers. Specifically, in perirenal WAT of HF offspring, where the leptin promoter showed a decrease in 5mC and an increase in 5hmC in perirenal WAT of HF offspring, suggesting that this specific leptin regulation may be associated with active DNA demethylation (120). Another study, using a similar approach, confirmed that reduced PPARγ2 expression in perirenal adipose tissue of HF offspring was associated with persistent hypermethylation (121). In light of this, could promoting the degree of DNA demethylation of adipose-associated genes in HF offspring be an effective way to ameliorate the transcriptional repression of hypermethylation? What role do key enzymes regulating DNA methylation/demethylation, such as DNMTs and TET proteins, play in this epigenetic mechanism? These questions warrant further investigation to clarify their biological and translational implications.

## 5 Concluding remarks

Epigenetic mechanisms exert profound influences on biological processes, with DNA methylation and demethylation serving as central regulatory processes in human biology and disease pathogenesis. Adipocyte biology, a key biological hub in metabolic diseases such as obesity, diabetes, and non-alcoholic fatty liver disease, is profoundly shaped by epigenetic mechanisms (**Table 1**). DNA demethylation reverses gene hypermethylation or abnormal methylation status, thereby counteracting DNA methylation-mediated gene silencing. This process is widely involved in adipocyte biology by promoting the expression of specific genes. DNMT enzymes are central regulators of DNA methylation and passive demethylation. In adipocyte biology, active DNMTs stimulate adipogenesis and inflammatory responses in adults with obesity, whereas inhibition of DNMT activity may suppress these processes. However, a seemingly paradoxical phenomenon is that knocking out DNMT exacerbates lipid accumulation and the progression of obesity. Although part of this phenomenon is due to DNMT acting on different target genes, it is not yet known whether it is related to the level of active DNMT enzymes. In adipocyte biology, TET-mediated active DNA demethylation mechanisms exhibit prominent multi-pathway regulatory effects. TET proteins regulate adipocyte biological processes either autonomously or through demethylation, promoting PPARα and PPARγ gene expression to regulate adipogenesis, while also having a bidirectional regulation on leptin production, and are regulated by cofactors. Additionally, the BER pathway, as a link in the classical TET-TDG-BER pathway, TDG interferes with the lipogenesis process in the same way as MBD4.

**Table 1:** The specific action mechanisms of DNMTs and TETs in adipocyte biology.

Enzymes	Classification	Mechanisms of action	Biological process	Reference
DNMTs	DNMT1	- Maintenance of DNA methylation - Inhibition of lipocalin expression - Activated by high glucose/oxysterols	- Bidirectional lipogenesis inhibition/promotion - Gene overexpression induces an inflammatory response - Gene silencing accelerates adipocyte differentiation/promotes lipid accumulation/resists increased adiposity	(39, 8, 48, 49, 51, 42)
	DNMT3A	- mediate *de novo* methylation - Maintenance of DNA methylation - Induction of leptin CPG methylation	- Gene overexpression inhibits adipocyte differentiation, lipogenesis, and induction of inflammatory responses - Gene deletion induces obesity/insulin resistance in old age	(40, 41, 8, 44, 54)
	DNMT3B	- Synergistic mediation of *de novo* methylation and maintenance of methylation	- Knockouts lead to lower energy expenditure	(55)
TETs	TET1	- Mediates gradual oxidation of 5mC - Regulation of PPARγ expression - Hydroxymethylation activates PPARα	- Knockout promotes fatty acid uptake - Bidirectional regulation of lipogenesis (inhibition of thermogenesis or promotion of lipid metabolism) - Promote fatty acid oxidation	(76, 81, 89, 92, 86, 94)
	TET2	- Catalytic-dependent/non-dependent promotion of PPARγ transcriptional activity - Mediates C-Maf-induced protein methylation - Inhibited by leptin	- Regulation of endothelial cell gene expression - Gene knockdown blocks lipogenesis - Regulating progress in NAFLD - Gene deficiency targeting leptin improves obesity and modulates insulin sensitivity	(78, 84, 89, 90, 91, 93)
	TET3	- Autoregulation of lipogenesis cells - Erase DNA methylation (e.g. C/EBPδ binding sites) - Negative regulation of Agrp expression	- Promote white fat deposition, adipocyte differentiation - Modulation of appetite in obesity-related centers	(77, 80, 102, 103)

While the mechanisms and biological significance of DNA demethylation in adipocyte biology have been explored through multiple approaches, existing studies leave several key questions unresolved. Firstly, the role of DNMTi in adipocyte biology and lipid metabolism-related disorders is far less well-explored compared to cancer, with current research scope being relatively narrow. In particular, in the available studies, targeting 5-Ac in adipocytes has identified genomic loci of DNA methylation, but the mechanisms governing its removal remain to be elucidated. Secondly, TET2 has been the focus of more targeted studies owing to the unique of its structural domains. While many of these studies have reported TET2’s role in regulating the PPARγ gene, future research is needed to fully characterize the broad impact of TET2-dependent transcription on PPARγ genomic targets. TET2 differs structurally from TET1 and TET3 and exhibits specific binding to transcription factors in the demethylation pathway. This suggests that targeting TET2-transcription factor interactions represents a safer and more specific therapeutic strategy. Most critically, current research on DNMT-mediated passive and TET-mediated active DNA demethylation mechanisms in adipocyte biology is predominantly focused on *in vitro* and animal models, lacking robust clinical evidence to validate these findings. In summary, despite over a decade of investigation, the molecular mechanisms of DNA demethylation in adipocyte biology remain incompletely understood. Their functional implications likely extend beyond our current knowledge, with future studies poised to reveal additional critical insights into their roles in metabolic health and disease.

## References

[1] Mattei AL Bailly N Meissner A. DNA methylation: a historical perspective. Trends Genet. (2022) 38:676–707. doi: 10.1016/j.tig.2022.03.010 35504755

[2] Robertson KD. DNA methylation and human disease. Nat Rev Genet. (2005) 6:597–610. doi: 10.1038/nrg1655 16136652

[3] Weber M Schübeler D. Genomic patterns of DNA methylation: targets and function of an epigenetic mark. Curr Opin Cell Biol. (2007) 19:273–80. doi: 10.1016/j.ceb.2007.04.011 17466503

[4] Moore LD Le T Fan GJN. DNA methylation and its basic function. Neuropsychopharmacology (2013) 38:23–38. doi: 10.1038/npp.2012.112 22781841 PMC3521964

[5] Schübeler D. Function and information content of DNA methylation. Nature. (2015) 517:321–6. doi: 10.1038/nature14192 25592537

[6] Campión J MILAGRO FI Martínez JA. Individuality and epigenetics in obesity. Obes Rev. (2009) 10:383–92. doi: 10.1111/j.1467-789X.2009.00595.x 19413700

[7] Sakamoto H Kogo Y Ohgane J Hattori N Yagi S Tanaka S. Sequential changes in genome-wide DNA methylation status during adipocyte differentiation. Biochem Biophys Res Commun. (2008) 366:360–6. doi: 10.1016/j.bbrc.2007.11.137 18062916

[8] Yang X Wu R Shan W Yu L Xue B Shi H. DNA methylation biphasically regulates 3T3-L1 preadipocyte differentiation. Mol Endocrinol. (2016) 30:677–87. doi: 10.1210/me.2015-1135 PMC4884341 27144289

[9] McAllan L Baranasic D Villicaña S Brown S Zhang W Lehne B. Integrative genomic analyses in adipocytes implicate DNA methylation in human obesity and diabetes. Nat Commun. (2023) 14:2784. doi: 10.1038/s41467-023-38439-z 37188674 PMC10185556

[10] Rosen ED MacDougald OA. Adipocyte differentiation from the inside out. Nat Rev Mol Cell Biol. (2006) 7:885–96. doi: 10.1038/nrm2066 17139329

[11] Wang W Seale P. Control of brown and beige fat development. Nat Rev Mol Cell Biol. (2016) 17:691–702. doi: 10.1038/nrm.2016.96 27552974 PMC5627770

[12] Wu J Boström P Sparks LM Ye L Choi JH Giang AH. Beige adipocytes are a distinct type of thermogenic fat cell in mouse and human. Cell. (2012) 150:366–76. doi: 10.1016/j.cell.2012.05.016 PMC3402601 22796012

[13] Jeon YG Kim YY Lee G Kim JB. Physiological and pathological roles of lipogenesis. Nat Metab. (2023) 5:735–59. doi: 10.1038/s42255-023-00786-y 37142787

[14] Liang J Jia Y Yu H Yan H Shen Q Xu Y. 5-Aza-2’-deoxycytidine regulates white adipocyte browning by modulating miRNA-133a/Prdm16. Metabolites. (2022) 12:1131. doi: 10.3390/metabo12111131 36422269 PMC9695087

[15] Dunn J Qiu H Kim S Jjingo D Hoffman R Kim CW. Flow-dependent epigenetic DNA methylation regulates endothelial gene expression and atherosclerosis. J Clin Invest. (2014) 124:3187–99. doi: 10.1172/JCI74792 PMC4071393 24865430

[16] Yu HL Dong S Gao LF Li L Xi YD Ma WW. Global DNA methylation was changed by a maternal high-lipid, high-energy diet during gestation and lactation in male adult mice liver. Br J Nutr. (2015) 113:1032–9. doi: 10.1017/S0007114515000252 25778733

[17] Seki Y Suzuki M Guo X Glenn AS Vuguin PM Fiallo A. *In utero* exposure to a high-fat diet programs hepatic hypermethylation and gene dysregulation and development of metabolic syndrome in male mice. Endocrinology. (2017) 158:2860–72. doi: 10.1210/en.2017-00334 PMC5659663 28911167

[18] Wilson AS Power BE Molloy PL. DNA hypomethylation and human diseases. Biochim Biophys Acta. (2007) 1775:138–62. doi: 10.1016/j.bbcan.2006.08.007 17045745

[19] Guo W Zhang KM Tu K Li YX Zhu L Xiao HS. Adipogenesis licensing and execution are disparately linked to cell proliferation. Cell Res. (2009) 19:216–23. doi: 10.1038/cr.2008.319 19065151

[20] Razin A Webb C Szyf M YIsraeli J Rosenthal A Naveh-Many T. Variations in DNA methylation during mouse cell differentiation *in vivo* and in *vitro* . Proc Natl Acad Sci U.S.A. (1984) 81:2275–9. doi: 10.1073/pnas.81.8.2275 PMC345041 6585800

[21] Seki Y Hayashi K Itoh K Mizugaki M Saitou M Matsui Y. Extensive and orderly reprogramming of genome-wide chromatin modifications associated with specification and early development of germ cells in mice. Dev Biol. (2005) 278:440–58. doi: 10.1016/j.ydbio.2004.11.025 15680362

[22] Kurimoto K Yabuta Y Ohinata Y Shigeta M Yamanaka K Saitou M. Complex genome-wide transcription dynamics orchestrated by Blimp1 for the specification of the germ cell lineage in mice. Genes Dev. (2008) 22:1617–35. doi: 10.1101/gad.1649908 PMC2428060 18559478

[23] Yamanaka S Blau HM. Nuclear reprogramming to a pluripotent state by three approaches. Nature. (2010) 465:704–12. doi: 10.1038/nature09229 PMC2901154 20535199

[24] Gehring M Reik W Henikoff S. DNA demethylation by DNA repair. Trends Genet. (2009) 25:82–90. doi: 10.1016/j.tig.2008.12.001 19144439

[25] Gjerset RA Martin DW Jr. Presence of a DNA demethylating activity in the nucleus of murine erythroleukemic cells. J Biol Chem. (1982) 257:8581–3. doi: 10.1016/S0021-9258(18)34161-9 7047522

[26] Rougier N Bourc’his D Gomes DM Niveleau A Plachot M Pàldi A. Chromosome methylation patterns during mammalian preimplantation development. Genes Dev. (1998) 12:2108–13. doi: 10.1101/gad.12.14.2108 PMC317005 9679055

[27] Tahiliani M Koh KP Shen Y Pastor WA Bandukwala H Brudno Y. Conversion of 5-methylcytosine to 5-hydroxymethylcytosine in mammalian DNA by MLL partner TET1. Science. (2009) 324:930–5. doi: 10.1126/science.1170116 PMC2715015 19372391

[28] Bhutani N Brady JJ Damian M Sacco A Corbel SY Blau HM. Reprogramming towards pluripotency requires AID-dependent DNA demethylation. Nature. (2010) 463:1042–7. doi: 10.1038/nature08752 PMC2906123 20027182

[29] Ito S D’Alessio AC Taranova OV Hong K Sowers LC Zhang Y. Role of Tet proteins in 5mC to 5hmC conversion, ES-cell self-renewal and inner cell mass specification. Nature. (2010) 466:1129–33. doi: 10.1038/nature09303 PMC3491567 20639862

[30] Cortellino S Xu J Sannai M Moore R Caretti E Cigliano A. Thymine DNA glycosylase is essential for active DNA demethylation by linked deamination-base excision repair. Cell. (2011) 146:67–79. doi: 10.1016/j.cell.2011.06.020 21722948 PMC3230223

[31] Ito S Shen L Dai Q Wu SC Collins LB Swenberg JA. Tet proteins can convert 5-methylcytosine to 5-formylcytosine and 5-carboxylcytosine. Science. (2011) 333:1300–3. doi: 10.1126/science.1210597 PMC3495246 21778364

[32] Schutsky EK Nabel CS Davis AKF Denizio JE Kohli RM. APOBEC3A efficiently deaminates methylated, but not TET-oxidized, cytosine bases in DNA. Nucleic Acids Res. (2017) 45:7655–65. doi: 10.1093/nar/gkx345 PMC5570014 28472485

[33] Feng Y Chen JJ Xie NB Ding JH You XJ Tao WB. Direct decarboxylation of ten-eleven translocation-produced 5-carboxylcytosine in mammalian genomes forms a new mechanism for active DNA demethylation. Chem Sci. (2021) 12:11322–9. doi: 10.1039/D1SC02161C PMC8409474 34567494

[34] Korytiaková E Kamińska E Müller M Carell T. Deformylation of 5-formylcytidine in different cell types. Angew Chem Int Ed Engl. (2021) 60:16869–73. doi: 10.1002/anie.202107089 PMC8362038 34110681

[35] Nabel CS Jia H Ye Y Shen L Goldschmidt HL Stivers JT. AID/APOBEC deaminases disfavor modified cytosines implicated in DNA demethylation. Nat Chem Biol. (2012) 8:751–8. doi: 10.1038/nchembio.1042 PMC3427411 22772155

[36] Zhang X Zhang Y Wang C Wang X. TET (Ten-eleven translocation) family proteins: structure, biological functions and applications. Signal Transduct Target Ther. (2023) 8:297. doi: 10.1038/s41392-023-01537-x 37563110 PMC10415333

[37] Lyko F. The DNA methyltransferase family: a versatile toolkit for epigenetic regulation. Nat Rev Genet. (2018) 19:81–92. doi: 10.1038/nrg.2017.80 29033456

[38] Okano M Bell DW Haber DA Li E. DNA methyltransferases Dnmt3a and Dnmt3b are essential for *de novo* methylation and mammalian development. Cell. (1999) 99:247–57. doi: 10.1016/S0092-8674(00)81656-6 10555141

[39] Goll MG Bestor TH. Eukaryotic cytosine methyltransferases. Annu Rev Biochem. (2005) 74:481–514. doi: 10.1146/annurev.biochem.74.010904.153721 15952895

[40] Jones PA Liang G. Rethinking how DNA methylation patterns are maintained. Nat Rev Genet. (2009) 10:805–11. doi: 10.1038/nrg2651 PMC2848124 19789556

[41] Kamei Y Suganami T Ehara T Kanai S Hayashi K Yamamoto Y. Increased expression of DNA methyltransferase 3a in obese adipose tissue: studies with transgenic mice. Obes (Silver Spring). (2010) 18:314–21. doi: 10.1038/oby.2009.246 19680236

[42] Londoño Gentile T Lu C Lodato PM Tse S Olejniczak SH Witze ES. DNMT1 is regulated by ATP-citrate lyase and maintains methylation patterns during adipocyte differentiation. Mol Cell Biol. (2013) 33:3864–78. doi: 10.1128/MCB.01495-12 PMC3811875 23897429

[43] Qimuge N He Z Qin J Sun Y Wang X Yu T. Overexpression of DNMT3A promotes proliferation and inhibits differentiation of porcine intramuscular preadipocytes by methylating p21 and PPARg promoters. Gene. (2019) 696:54–62. doi: 10.1016/j.gene.2019.02.029 30772521

[44] Xia L Wang C Lu Y Fan C Ding X Fu H. Time-specific changes in DNA methyltransferases associated with the leptin promoter during the development of obesity. Nutr Hosp. (2014) 30:1248–55. doi: 10.3305/nh.2014.30.6.7843 25433105

[45] Parrillo L Costa V Raciti GA Longo M Spinelli R Esposito R. Hoxa5 undergoes dynamic DNA methylation and transcriptional repression in the adipose tissue of mice exposed to high-fat diet. Int J Obes (Lond). (2016) 40:929–37. doi: 10.1038/ijo.2016.36 26980478

[46] Sukur G Uysal F Cinar O. High-fat diet induced obesity alters Dnmt1 and Dnmt3a levels and global DNA methylation in mouse ovary and testis. Histochem Cell Biol. (2023) 159:339–52. doi: 10.1007/s00418-022-02173-2 36624173

[47] Cierzniak A Pawelka D Kaliszewski K Rudnicki J Dobosz T Malodobra-Mazur M. DNA methylation in adipocytes from visceral and subcutaneous adipose tissue influences insulin-signaling gene expression in obese individuals. Int J Obes (Lond). (2021) 45:650–8. doi: 10.1038/s41366-020-00729-7 33414486

[48] Deng Y Qiu T Zhang M Wu J Zhang X Wang J. High level of palmitic acid induced over-expressed methyltransferase inhibits anti-inflammation factor KLF4 expression in obese status. Inflammation. (2020) 43:821–32. doi: 10.1007/s10753-019-01168-x 31900830

[49] Kim AY Park YJ Pan X Shin KC Kwak SH Bassas AF. Obesity-induced DNA hypermethylation of the adiponectin gene mediates insulin resistance. Nat Commun. (2015) 6:7585. doi: 10.1038/ncomms8585 26139044 PMC4506505

[50] Acosta JR Joost S Karlsson K Ehrlund A Li X Aouadi M. Single cell transcriptomics suggest that human adipocyte progenitor cells constitute a homogeneous cell population (2017). Available online at: https://adiposetissue.org/celltype (Accessed May 12, 2025). 10.1186/s13287-017-0701-4 PMC5678572 29116032

[51] Wang Y Chen L Pandak WM Heuman D Hylemon PB Ren S. High glucose induces lipid accumulation via 25-hydroxycholesterol DNA-cpG methylation. iScience. (2020) 23:101102. doi: 10.1016/j.isci.2020.101102 32408171 PMC7225732

[52] Lee HH An SM Ye BJ Lee JH Yoo EJ Jeong GW. TonEBP/NFAT5 promotes obesity and insulin resistance by epigenetic suppression of white adipose tissue beiging. Nat Commun. (2019) 10:3536. doi: 10.1038/s41467-019-11302-w 31387996 PMC6684655

[53] Park YJ Lee S Lim S Nahmgoong H Ji Y Huh JY. DNMT1 maintains metabolic fitness of adipocytes through acting as an epigenetic safeguard of mitochondrial dynamics. Proc Natl Acad Sci U.S.A. (2021) 118:e2021073118. doi: 10.1073/pnas.2021073118 33836591 PMC7980432

[54] Tovy A Reyes JM Zhang L Huang YH Rosas C Daquinag AC. Constitutive loss of DNMT3A causes morbid obesity through misregulation of adipogenesis. Elife. (2022) 11:e72359. doi: 10.7554/eLife.72359 35635747 PMC9150890

[55] Wang S Cao Q Cui X Jing J Li F Shi H. Dnmt3b deficiency in myf5(+)-brown fat precursor cells promotes obesity in female mice. Biomolecules. (2021) 11:1087. doi: 10.3390/biom11081087 34439754 PMC8393658

[56] Reyes JM Tovy A Zhang L Bortoletto AS Rosas C Chen CW. Hematologic DNMT3A reduction and high-fat diet synergize to promote weight gain and tissue inflammation. iScience. (2024) 27:109122. doi: 10.1016/j.isci.2024.109122 38414863 PMC10897855

[57] Uddin MG Fandy TE. DNA methylation inhibitors: Retrospective and perspective view. Adv Cancer Res. (2021) 152:205–23. doi: 10.1016/bs.acr.2021.03.007 PMC10275377 34353438

[58] Kantarjian H Issa JP Rosenfeld CS Bennett JM Albitar M Dipersio J. Decitabine improves patient outcomes in myelodysplastic syndromes: results of a phase III randomized study. Cancer. (2006) 106:1794–803. doi: 10.1002/cncr.v106:8 16532500

[59] Christman JK. 5-Azacytidine and 5-aza-2’-deoxycytidine as inhibitors of DNA methylation: mechanistic studies and their implications for cancer therapy. Oncogene. (2002) 21:5483–95. doi: 10.1038/sj.onc.1205699 12154409

[60] Flores-Sierra JJ Muciño-Arellano MDR Romo-Morales GDC Sánchez-Palafox JE Correa-Navarro VA Colín-Castelán D. The DNA methyltransferase inhibitor decitabine blunts the response to a high-animal fat and protein diet in mice. J Lipid Res. (2024) 65:100586. doi: 10.1016/j.jlr.2024.100586 38942113 PMC11325794

[61] Hasani S Javeri A Asadi A Fakhr Taha M. Cardiac Differentiation of Adipose Tissue-Derived Stem Cells Is Driven by BMP4 and bFGF but Counteracted by 5-Azacytidine and Valproic Acid. Cell J. (2020) 22:273–82. doi: 10.22074/cellj.2020.6582 PMC6947007 31863652

[62] Poirier S Samami S Mamarbachi M Demers A Chang TY Vance DE. The epigenetic drug 5-azacytidine interferes with cholesterol and lipid metabolism. J Biol Chem. (2014) 289:18736–51. doi: 10.1074/jbc.M114.563650 PMC4081918 24855646

[63] Kuroda M Tominaga A Nakagawa K Nishiguchi M Sebe M Miyatake Y. DNA methylation suppresses leptin gene in 3T3-L1 adipocytes. PloS One. (2016) 11:e0160532. doi: 10.1371/journal.pone.0160532 27494408 PMC4975473

[64] Marchi M Lisi S Curcio M Barbuti S Piaggi P Ceccarini G. Human leptin tissue distribution, but not weight loss-dependent change in expression, is associated with methylation of its promoter. Epigenetics. (2011) 6:1198–206. doi: 10.4161/epi.6.10.16600 PMC3225841 21931275

[65] Tang J Pulliam N Özeş A Buechlein A Ding N Keer H. Epigenetic targeting of adipocytes inhibits high-grade serous ovarian cancer cell migration and invasion. Mol Cancer Res. (2018) 16:1226–40. doi: 10.1158/1541-7786.MCR-17-0406 PMC6072573 29759990

[66] Pant R Kabeer SW Sharma S Kumar V Patra D Pal D. Pharmacological inhibition of DNMT1 restores macrophage autophagy and M2 polarization in Western diet-induced nonalcoholic fatty liver disease. J Biol Chem. (2023) 299:104779. doi: 10.1016/j.jbc.2023.104779 37142224 PMC10248527

[67] Gao J Cheng Y Hao H Yin Y Xue J Zhang Q. Decitabine assists umbilical cord-derived mesenchymal stem cells in improving glucose homeostasis by modulating macrophage polarization in type 2 diabetic mice. Stem Cell Res Ther. (2019) 10:259. doi: 10.1186/s13287-019-1338-2 31426846 PMC6700792

[68] Ke ZH Pan JX Jin LY Xu HY Yu TT Ullah K. Bisphenol A exposure may induce hepatic lipid accumulation via reprogramming the DNA methylation patterns of genes involved in lipid metabolism. Sci Rep. (2016) 6:31331. doi: 10.1038/srep31331 27502578 PMC4977563

[69] Lorsbach RB Moore J Mathew S Raimondi SC Mukatira ST Downing JR. TET1, a member of a novel protein family, is fused to MLL in acute myeloid leukemia containing the t(10;11)(q22;q23). Leukemia. (2003) 17:637–41. doi: 10.1038/sj.leu.2402834 12646957

[70] Ono R Taki T Taketani T Taniwaki M Kobayashi H Hayashi Y. LCX, leukemia-associated protein with a CXXC domain, is fused to MLL in acute myeloid leukemia with trilineage dysplasia having t(10;11)(q22;q23). Cancer Res. (2002) 62:4075–80.Available at: https://aacrjournals.org/cancerres/article/62/14/4075/508997/LCX-Leukemia-associated-Protein-with-a-CXXC-Domain?searchresult=1. 12124344

[71] Wu X Zhang Y. TET-mediated active DNA demethylation: mechanism, function and beyond. Nat Rev Genet. (2017) 18:517–34. doi: 10.1038/nrg.2017.33 28555658

[72] Sardina JL Collombet S Tian TV Gómez A Di Stefano B Berenguer C. Transcription factors drive tet2-mediated enhancer demethylation to reprogram cell fate. Cell Stem Cell. (2018) 23:727–741.e9. doi: 10.1016/j.stem.2018.08.016 30220521

[73] Pastor WA Aravind L Rao A. TETonic shift: biological roles of TET proteins in DNA demethylation and transcription. Nat Rev Mol Cell Biol. (2013) 14:341–56. doi: 10.1038/nrm3589 PMC3804139 23698584

[74] Wang D Wu W Callen E Pavani R Zolnerowich N Kodali S. Active DNA demethylation promotes cell fate specification and the DNA damage response. Science. (2022) 378:983–9. doi: 10.1126/science.add9838 PMC10196940 36454826

[75] Scourzic L Mouly E Bernard OA. TET proteins and the control of cytosine demethylation in cancer. Genome Med. (2015) 7:9. doi: 10.1186/s13073-015-0134-6 25632305 PMC4308928

[76] Damal Villivalam S You D Kim J Lim HW Xiao H Zushin PH. TET1 is a beige adipocyte-selective epigenetic suppressor of thermogenesis. Nat Commun. (2020) 11:4313. doi: 10.1038/s41467-020-18054-y 32855402 PMC7453011

[77] Jung BC You D Lee I Li D Schill RL Ma K. TET3 plays a critical role in white adipose development and diet-induced remodeling. Cell Rep. (2023) 42:113196. doi: 10.1016/j.celrep.2023.113196 37777963 PMC10763978

[78] Hou Y Zhang Z Wang Y Gao T Liu X Tang T. 5mC profiling characterized TET2 as an anti-adipogenic demethylase. Gene. (2020) 733:144265. doi: 10.1016/j.gene.2019.144265 31805318

[79] Cakouros D Hemming S Gronthos K Liu R Zannettino A Shi S. Specific functions of TET1 and TET2 in regulating mesenchymal cell lineage determination. Epigenet Chromatin. (2019) 12:3. doi: 10.1186/s13072-018-0247-4 PMC6317244 30606231

[80] Park J Lee DH Ham S Oh J Noh JR Lee YK. Targeted erasure of DNA methylation by TET3 drives adipogenic reprogramming and differentiation. Nat Metab. (2022) 4:918–31. doi: 10.1038/s42255-022-00597-7 35788760

[81] Yuan Y Liu C Chen X Sun Y Xiong M Fan Y. Vitamin C inhibits the metabolic changes induced by tet1 insufficiency under high fat diet stress. Mol Nutr Food Res. (2021) 65:e2100417. doi: 10.1002/mnfr.202100417 34129274

[82] Qian H Zhao J Yang X Wu S An Y Qu Y. TET1 promotes RXRα expression and adipogenesis through DNA demethylation. Biochim Biophys Acta Mol Cell Biol Lipids. (2021) 1866:158919. doi: 10.1016/j.bbalip.2021.158919 33684567

[83] Byun S Lee CH Jeong H Kim H Kwon HM Park S. Loss of adipose TET proteins enhances β-adrenergic responses and protects against obesity by epigenetic regulation of β3-AR expression. Proc Natl Acad Sci U.S.A. (2022) 119:e2205626119. doi: 10.1073/pnas.2205626119 35737830 PMC9245707

[84] Shi Y Huang X Zeng Y Zhai M Yao H Liu C. Endothelial TET2 regulates the white adipose browning and metabolism via fatty acid oxidation in obesity. Redox Biol. (2024) 69:103013. doi: 10.1016/j.redox.2023.103013 38168657 PMC10797209

[85] Liu B Xie D Huang X Jin S Dai Y Sun X. Skeletal muscle TET3 promotes insulin resistance through destabilisation of PGC-1α. Diabetologia. (2024) 67:724–37. doi: 10.1007/s00125-023-06073-5 PMC10904493 38216792

[86] Montaigne D Butruille L Staels B. PPAR control of metabolism and cardiovascular functions. Nat Rev Cardiol. (2021) 18:809–23. doi: 10.1038/s41569-021-00569-6 34127848

[87] Gross B Pawlak M Lefebvre P Staels B. PPARs in obesity-induced T2DM, dyslipidaemia and NAFLD. Nat Rev Endocrinol. (2017) 13:36–49. doi: 10.1038/nrendo.2016.135 27636730

[88] Sérandour AA Avner S Oger F Bizot M Percevault F Lucchetti-Miganeh C. Dynamic hydroxymethylation of deoxyribonucleic acid marks differentiation-associated enhancers. Nucleic Acids Res. (2012) 40:8255–65. doi: 10.1093/nar/gks595 PMC3458548 22730288

[89] Yoo Y Park JH Weigel C Liesenfeld DB Weichenhan D Plass C. TET-mediated hydroxymethylcytosine at the Pparγ locus is required for initiation of adipogenic differentiation. Int J Obes (Lond). (2017) 41:652–9. doi: 10.1038/ijo.2017.8 28100914

[90] Bian F Ma X Villivalam SD You D Choy LR Paladugu A. TET2 facilitates PPARγ agonist-mediated gene regulation and insulin sensitization in adipocytes. Metabolism. (2018) 89:39–47. doi: 10.1016/j.metabol.2018.08.006 30193945 PMC6221917

[91] Liu Y He T Li Z Sun Z Wang S Shen H. TET2 is recruited by CREB to promote Cebpb, Cebpa, and Pparg transcription by facilitating hydroxymethylation during adipocyte differentiation. iScience. (2023) 26:108312. doi: 10.1016/j.isci.2023.108312 38026190 PMC10663734

[92] Wang J Zhang Y Zhuo Q Tseng Y Wang J Ma Y. TET1 promotes fatty acid oxidation and inhibits NAFLD progression by hydroxymethylation of PPARα promoter. Nutr Metab (Lond). (2020) 17:46. doi: 10.1186/s12986-020-00466-8 32577122 PMC7304222

[93] Lee J Song JH Park JH Chung MY Lee SH Jeon SB. Dnmt1/Tet2-mediated changes in Cmip methylation regulate the development of nonalcoholic fatty liver disease by controlling the Gbp2-Pparγ-CD36 axis. Exp Mol Med. (2023) 55:143–57. doi: 10.1038/s12276-022-00919-5 PMC9898513 36609599

[94] Mulla J Nagaoka K Cao K Cheng Z Bai X Zhang H. Inhibiting TET1 alleviates noneviatesnla liver disease progression by targeting lipid metabolic pathways. The FASEB Journal (2022), 36. doi: 10.1096/fasebj.2022.36.S1.0R520

[95] Zhang Y Proenca R Maffei M Barone M Leopold L Friedman JM. Positional cloning of the mouse obese gene and its human homologue. Nature. (1994) 372:425–32. doi: 10.1038/372425a0 7984236

[96] Schwartz MW Woods SC Porte D Jr. Seeley RJ Baskin DG. Central nervous system control of food intake. Nature. (2000) 404:661–71. doi: 10.1038/35007534 10766253

[97] Zhou Y Rui L. Leptin signaling and leptin resistance. Front Med. (2013) 7:207–22. doi: 10.1007/s11684-013-0263-5 PMC4069066 23580174

[98] Considine RV Considine EL Williams CJ Nyce MR Magosin SA Bauer TL. Evidence against either a premature stop codon or the absence of obese gene mRNA in human obesity. J Clin Invest. (1995) 95:2986–8. doi: 10.1172/JCI118007 PMC295988 7769141

[99] Shen W Wang C Xia L Fan C Dong H Deckelbaum RJ. Epigenetic modification of the leptin promoter in diet-induced obese mice and the effects of N-3 polyunsaturated fatty acids. Sci Rep. (2014) 4:5282. doi: 10.1038/srep05282 24923522 PMC5381469

[100] Deem JD Faber CL Morton GJ. AgRP neurons: Regulators of feeding, energy expenditure, and behavior. FEBS J. (2022) 289:2362–81. doi: 10.1111/febs.v289.8 PMC9040143 34469623

[101] Cowley MA Smart JL Rubinstein M Cerdán MG Diano S Horvath TL. Leptin activates anorexigenic POMC neurons through a neural network in the arcuate nucleus. Nature. (2001) 411:480–4. doi: 10.1038/35078085 11373681

[102] Xie D Stutz B Li F Chen F Lv H Sestan-Pesa M. TET3 epigenetically controls feeding and stress response behaviors via AGRP neurons. J Clin Invest. (2022) 132:e162365. doi: 10.1172/JCI162365 36189793 PMC9525119

[103] Zeng Q Song J Sun X Wang D Liao X Ding Y. A negative feedback loop between TET2 and leptin in adipocyte regulates body weight. Nat Commun. (2024) 15:2825. doi: 10.1038/s41467-024-46783-x 38561362 PMC10985112

[104] Martinez S Hausinger RP. Catalytic mechanisms of Fe(II)- and 2-oxoglutarate-dependent oxygenases. J Biol Chem. (2015) 290:20702–11. doi: 10.1074/jbc.R115.648691 PMC4543632 26152721

[105] Suzuki T Komatsu T Shibata H Tanioka A Vargas D Kawabata-Iwakawa R. Crucial role of iron in epigenetic rewriting during adipocyte differentiation mediated by JMJD1A and TET2 activity. Nucleic Acids Res. (2023) 51:6120–42. doi: 10.1093/nar/gkad342 PMC10325906 37158274

[106] Yin R Mao SQ Zhao B Chong Z Yang Y Zhao C. Ascorbic acid enhances Tet-mediated 5-methylcytosine oxidation and promotes DNA demethylation in mammals. J Am Chem Soc. (2013) 135:10396–403. doi: 10.1021/ja4028346 23768208

[107] Chen J Guo L Zhang L Wu H Yang J Liu H. Vitamin C modulates TET1 function during somatic cell reprogramming. Nat Genet. (2013) 45:1504–9. doi: 10.1038/ng.2807 24162740

[108] Krokan HE Bjørås M. Base excision repair. Cold Spring Harb Perspect Biol. (2013) 5:a012583. doi: 10.1101/cshperspect.a012583 23545420 PMC3683898

[109] Pidugu LS Flowers JW Coey CT Pozharski E Greenberg MM Drohat AC. Structural basis for excision of 5-formylcytosine by thymine DNA glycosylase. Biochemistry. (2016) 55:6205–8. doi: 10.1021/acs.biochem.6b00982 PMC5148694 27805810

[110] Zhang L Lu X Lu J Liang H Dai Q Xu GL. Thymine DNA glycosylase specifically recognizes 5-carboxylcytosine-modified DNA. Nat Chem Biol. (2012) 8:328–30. doi: 10.1038/nchembio.914 PMC3307914 22327402

[111] Maiti A Drohat AC. Thymine DNA glycosylase can rapidly excise 5-formylcytosine and 5-carboxylcytosine: potential implications for active demethylation of CpG sites. J Biol Chem. (2011) 286:35334–8. doi: 10.1074/jbc.C111.284620 PMC3195571 21862836

[112] He YF Li BZ Li Z Liu P Wang Y Tang Q. Tet-mediated formation of 5-carboxylcytosine and its excision by TDG in mammalian DNA. Science. (2011) 333:1303–7. doi: 10.1126/science.1210944 PMC3462231 21817016

[113] Weber AR Krawczyk C Robertson AB Kuśnierczyk A Vågbø CB Schuermann D. Biochemical reconstitution of TET1-TDG-BER-dependent active DNA demethylation reveals a highly coordinated mechanism. Nat Commun. (2016) 7:10806. doi: 10.1038/ncomms10806 26932196 PMC4778062

[114] Zhang LJ Liu SY Zhu YN Gao Y Chen J Yuan B. Thymine DNA glycosylase gene knockdown can affect the differentiation of pig preadipocytes. Cell Physiol Biochem. (2016) 39:975–84. doi: 10.1159/000447805 27513857

[115] Pidugu LS Bright H Lin WJ Majumdar C Van Ostrand RP David SS. Structural insights into the mechanism of base excision by MBD4. J Mol Biol. (2021) 433:167097. doi: 10.1016/j.jmb.2021.167097 34107280 PMC8286355

[116] Hashimoto H Zhang X Cheng X. Excision of thymine and 5-hydroxymethyluracil by the MBD4 DNA glycosylase domain: structural basis and implications for active DNA demethylation. Nucleic Acids Res. (2012) 40:8276–84. doi: 10.1093/nar/gks628 PMC3458566 22740654

[117] Zhang LJ Zhu YN Gao Y Liu SY Zhai B Li CH. The MBD4 gene plays an important role in porcine adipocyte differentiation. Cell Physiol Biochem. (2014) 34:1216–26. doi: 10.1159/000366333 25277759

[118] Deshpande SSS Nemani H Balasinor NH. High fat diet-induced- and genetically inherited- obesity differential alters DNA demethylation pathways in the germline of adult male rats. Reprod Biol. (2021) 21:100532. doi: 10.1016/j.repbio.2021.100532 34246869

[119] Barker DJ. Developmental origins of adult health and disease. J Epidemiol Community Health. (2004) 58:114–5. doi: 10.1136/jech.58.2.114 PMC1732687 14729887

[120] Lecoutre S Oger F Pourpe C Butruille L Marousez L Dickes-Coopman A. Maternal obesity programs increased leptin gene expression in rat male offspring via epigenetic modifications in a depot-specific manner. Mol Metab. (2017) 6:922–30. doi: 10.1016/j.molmet.2017.05.010 PMC5518658 28752055

[121] Lecoutre S Pourpe C Butruille L Marousez L Laborie C Guinez C. Reduced PPARγ2 expression in adipose tissue of male rat offspring from obese dams is associated with epigenetic modifications. FASEB J. (2018) 32:2768–78. doi: 10.1096/fj.201700997R 29295860

